# Chances of late surgery in relation to length of wait lists

**DOI:** 10.1186/1472-6963-5-63

**Published:** 2005-09-26

**Authors:** Boris G Sobolev, Adrian R Levy, Lisa Kuramoto, Robert Hayden

**Affiliations:** 1 Department of Health Care and Epidemiology, University of British Columbia, Vancouver, Canada; 2 Centre for Clinical Epidemiology and Evaluation, Vancouver Coastal Health Research Institute, Vancouver, Canada; 3 Centre for Health Evaluation and Outcome Sciences, St. Paul's Hospital, Vancouver, Canada; 4 Department of Surgery, Royal Columbian Hospital, New Westminster, Canada

## Abstract

**Background:**

The proportion of patients who undergo surgery within a clinically safe time is an important performance indicator in health systems that use wait lists to manage access to care. However, little is known about chances of on-time surgery according to variations in existing demand. We sought to determine what proportion of patients have had late coronary bypass surgery after registration on wait lists of different size in a network of hospitals with uniform standards for timing of surgery.

**Methods:**

Using records from a population-based registry, we studied wait-list times prospectively collected in a cohort of patients registered on wait lists for coronary artery bypass grafting procedures. We compared the number of weeks from registration to surgery against target access times established for three urgency groups. The chances of undergoing surgery within target time have been evaluated in relation to wait-list size at registration and the number of surgeries performed without registration on a wait list.

**Results:**

In 1991–2001, two in three patients were at risk of late surgery when registered on wait lists for isolated coronary bypass procedures in British Columbia, Canada. Although urgent patients had never seen a wait list with clearance time exceeding one week, the odds of on-time surgery were reduced by 25%, odds ratio [OR] = 0.75 (95% confidence interval [CI] 0.65–0.87) for every additional operation performed without registration on a list. When the wait list at registration required a clearance time of over one month, semi-urgent patients had 51% lower odds of on-time surgery as compared to lists with clearance time less than one week, OR = 0.49 (95%CI 0.41–0.60), after adjustment for age, sex, comorbidity, calendar period, hospital and week on the list. In the non-urgent group, the odds were 69% lower, OR = 0.31 (95%CI 0.20–0.47). Every time an operation in the same hospital was performed without registration on a wait list, the odds of on-time surgery for listed patients were reduced by 7%, OR = 0.93 (95%CI 0.91–0.95) in the semi-urgent group, and by 10%, OR = 0.90 (95%CI 0.87–0.94), in the non-urgent group.

**Conclusion:**

Chances of late surgery increase with the wait-list size for semi-urgent and non-urgent patients needing coronary bypass surgery. The weekly number of patients who move immediately from angiography to the operation without registration on a wait list reduced chances of surgery within target time in all urgency groups of listed patients. When advising patients who will be placed on the wait list about the expected time to treatment, hospital managers should take into account the current list size as well as the weekly number of patients who require CABG immediately after undergoing coronary angiography.

## Background

In health systems that provide universal access to care, efforts to contain costs for stand-by hospital capacity usually result in waiting lists for surgical procedures [1]. From clinical perspective, however, delay in necessary treatment due to surgical wait lists is a major concern [2,3]. Establishing a clinically appropriate time that patients can safely wait for the operation is generally perceived as a method to prevent adverse outcomes of delay [4]. For example, priority wait lists [5] are commonly used for queuing patients with coronary artery disease requiring bypass surgery based on the severity of condition [6,7].

The proportion of patients who undergo surgery within clinically acceptable time is an important performance indicator in health systems that use wait lists to manage access to care [8,9]. Describing variations in waiting times for coronary artery bypass surgery (CABG), Katz et al suggested that the wait-list size may be an important factor in delaying surgery [10]. Indeed, if there are patients on the list, then for a patient who just arrived to be admitted within a certain time all patients ahead must have been served. Sobolev et al performed an empirical analysis of a population-based registry and found that the length of queue at registration affected the time to elective surgery [11]. Surprisingly, little is known about how the list size at registration affects the chances of undergoing elective surgical procedures within acceptable time. The common concern for evaluation purposes is, therefore, whether one can accurately estimate the proportion of late surgeries without considering the length of a wait list.

In theory, queuing procedures should ensure access to care according to urgency of treatment if implemented uniformly across a health system [8]. However, the chances of admission for elective surgery within target time can be easily altered if surgical services experience an uneven influx of more urgent case [12]. In the Canadian province of British Columbia, there are two pathways to surgical revascularization: registration on a wait list, or direct admission after coronary angiography, as described in [11]. Patients presenting with symptoms of coronary artery disease are referred to cardiologist to assess the need for coronary revascularization. The cardiologist evaluates the coronary angiogram and decides on treatment. If coronary angioplasty is not indicated, then a cardiac surgeon is consulted to assess the patients' suitability for coronary bypass surgery. Following the consultation in which surgery is indicated, surgeons register on their wait lists patients who require and decide to undergo the operation. Alternatively, patients may be admitted to a hospital cardiac ward directly from the catheterization laboratory when urgent assessment is deemed necessary. If suitable for surgery, such patients remain in hospital until the operation.

The objective of this study was to determine the proportion of patients that have had late surgery after registration on surgical wait list of different sizes. To examine the association between the length of wait lists and timely access to elective CABG surgery, we used data on registrations and waiting times for elective coronary bypass surgery collected at a provincial cardiac surgery registry in BC. We also examined the relation between the number of surgeries performed without wait-list registration and the chances of surgery within recommended time among wait-listed patients.

## Methods

### Data sources

Data were taken from a population-based registry set up to capture the time of registration for surgery, the time of surgery or removal from wait lists without surgery, for all patients accepted for coronary bypass surgery in the four tertiary hospitals delivering adult cardiac care to residents of BC [15]. Offices of all cardiac surgeons weekly provide information to the registry on registrations for surgery, operations performed, waitlist reconciliation (removals), and discharge summaries. Coexisting medical conditions were identified in the BC Linked Health Database Hospital Separations File via a deterministic link with the registry records.

### Patients

There were 9,366 records of registration for isolated CABG added to the registry between January 1991 and December 2000. We excluded 147 records of patients who were emergency cases (44), were removed on the registration date (99), or who had missing operating room reports (4). The remaining 9,219 records had either the surgery date or the date and reason of removal from the list without surgery.

As patients who moved from angiography to surgery on an expedited basis were not added to the wait lists, they were not included in the analysis of wait-list times. These patients contributed to demand figures only.

### Urgency groups

All cardiac surgeons in BC use a common guideline for prioritizing patients and assigning a target time for surgery based on angina symptoms, affected coronary anatomy, and left ventricular function impairment as described elsewhere [15]. Each patient was classified as urgent if the suggested time to surgery was three days, semi-urgent if the time was six weeks, and non-urgent if the time was 12 weeks.

### Demand for surgery

For each calendar week during the study period, the demand for surgery was characterized by the existing list size and the number of direct admissions immediately after angiography. For each patient, the list size was a census of patients with higher or equal priority present at registration on the list in a hospital. Patients contributed one count to the list size for each week they remained on the list, except for the week of arrival. As operations are scheduled a week in advance, patients who underwent surgery are considered removed from the wait list in the week previous to their admission dates. The number of direct admissions was a weekly count of surgeries performed without wait-list registration.

### Comorbidity

We used diagnoses reported in discharge abstracts within one year prior to registration for coronary bypass surgery. Each patient was classified as 1) presenting with no co-existing conditions, 2) presenting with congestive heart failure, diabetes, chronic obstructive pulmonary disease, cancer or rheumatoid arthritis, or 3) presenting with other co-existing chronic conditions as defined in [16].

### Statistical analysis

#### Waiting time

Each patient had a waiting time computed as the number of calendar weeks between registration and surgery or removal for other reasons. The date at which a surgeon's office submits the operating room booking request for surgery served as the date of registration on the list. For procedures delayed beyond target access time, we studied the number of weeks to target time.

#### Study variables

The list size was categorized in relation to clearance time, that is, a hypothetical time within which the list will be cleared at a maximum weekly service capacity if there are no new arrivals. We divided the list size in four categories: 1) lists requiring less than a week of clearance time, 2) half a month, 3) a month, and 4) over one month. In three hospitals with the service capacity of 15 operations a week, the following numbers of patients on the list – 0 to 14; 15 to 29; 30 to 59; and over 60 – correspond to clearance time of a week, half a month, a month, over one month. In a hospital with the service capacity of 25 operations weeks, the same clearance times correspond to 0 to 24, 25 to 49, 50 to 99 and over 100 patients on the list. The weekly number of direct admissions was treated as a continuous variable.

#### Regression models

Primary outcome was admission to surgery within target access time. Primary comparisons were between wait-list size categories. To evaluate the effect of the list size, we estimated the odds ratios associated with list-size categories using discrete-time survival regression models for each urgency group [14]. In this service, scheduling patients for surgery has provided a weekly opportunity for admission to occur. Measured as the number of service scheduling cycles, waiting time is inherently discrete and is best measured as the number of new scheduling cycles from registration to admission or removal for other reasons. For this analysis, we performed a pooled analysis of binary regression models developed for each week on the list, treating weeks as ordered strata [13]. All cases removed from waiting lists without surgery or that exceeded target access time were treated as censored observations at one week after the target time. In multivariate analysis we adjusted for age, sex, comorbidity, period, the number of direct admissions and hospital.

We entered an indicator variable for each hospital in the models in order to obtain regression estimates for the study variables adjusted for possible variations in access management. Hospital 1 was coded as referent.

For the direct admissions, we interpret the odds ratio derived from the model as a change in the weekly odds of on-time surgery associated with one additional surgery performed immediately after angiography.

## Results

### Waiting outcomes

The baseline characteristics of registered patients are shown in Table 1. The most prevalent groups at registration were patients aged 60–69 (38%) and 70–79 (30%) years, men (82%), those registered in semi-urgent group (71%), those without major comorbidities (52%), and those registered in 1995–1996 (22%). Among the four hospitals, the majority of patients were registered at hospital 2 (34%). At registration most patients (43%) saw a list-size requiring one month of clearance time, whereas the minority (14%) required half a month.

**Table 1:** Characteristics of 9,219 subjects registered for isolated coronary artery bypass surgery in British Columbia 1991–2000

**Characteristic**	**N**	**(%)**
**Age group (yr)**		
<50	731	(7.9)
50–59	2006	(21.8)
60–69	3526	(38.2)
70–79	2764	(30.0)
≥80	192	(2.1)

**Sex**		
Women	1630	(17.7)
Men	7589	(82.3)

**Urgency at registration**		
Urgent	652	(7.1)
Semi-urgent	6553	(71.1)
Non-urgent	2014	(21.8)

**Major comorbidity at registration**		
None	4775	(51.8)
CHF, diabetes, COPD, rheumatoid arthritis, cancer	2435	(26.4)
Other conditions	2009	(21.8)

**Calendar period**		
1991–1992	1725	(18.7)
1993–1994	1889	(20.5)
1995–1996	1997	(21.7)
1997–1998	1888	(20.5)
1999–2000	1720	(18.7)

**Hospital ID at booking**		
1	1903	(20.6)
2	3137	(34.0)
3	2124	(23.0)
4	2055	(22.3)

**Wait-list size category**		
1 – <1 week	1502	(16.3)
2 – half month	1276	(13.8)
3 – 1 month	3954	(42.9)
4 – >1 month	2487	(27.0)

CHF – congestive heart failure, COPD – chronic obstructive pulmonary disease

Among those who was removed before or on a target access time (TAT), 2959 (93.6%) received surgery, 37 (1.2%) died, 64 (2.0%) continued with medical treatment, 31 (1.0%) declined surgery, 22 (0.7%) were transferred to another surgeon or hospital, and 47 (1.5%) were removed for other reasons (data not shown).

Among those who was removed after TAT, 5018 (82.2%) eventually underwent surgery, 55 (0.9%) died, 112 (1.9%) continued with medical treatment, 157 (2.6%) declined surgery, 77 (1.3%) were transferred to another surgeon or hospital, 165 (2.7%) were removed for other reasons, and 475 (7.8%) were still on the wait list at 52 weeks (data not shown).

Of the 652 urgent patients, 22 (3.4%) were removed from the wait list without surgery, and in 106 (16.3%) patients the urgency was downgraded to semi-urgent or non-urgent.

### Access to surgery in urgency groups

Overall, the proportion of patients who underwent surgery within the target access time was 32% (95% confidence interval [CI] 31–33%). The proportion varied significantly across urgency groups. Table 2 shows that among urgent, semi-urgent, and non-urgent patients, 21% (18–24%), 34% (33–35%), and 29% (27–31%) underwent surgery on time, respectively. The proportions of CABG performed within target time were similar in three hospitals ranging between 34 and 38%, with only 19% in hospital 3, Table 2.

**Table 2:** Probability of undergoing surgery within target time in relation to urgency, hospital, and wait-list size

**Characteristic**	**N**	**%**	**(95% CI)**
**Urgency at registration**			
Urgent	137	21.0	(17.9, 24.1)
Semi-urgent	2235	34.1	(33.0, 35.3)
Non-urgent	587	29.1	(27.2, 31.1)

**Hospital ID at booking**			
1	695	36.5	(34.4, 38.7)
2	1079	34.4	(32.7, 36.1)
3	405	19.1	(17.4, 20.7)
4	780	38.0	(35.9, 40.1)

**Wait-list size category**			
1 – <1 week	596	39.7	(37.2, 42.2)
2 – half month	454	35.6	(33.0, 38.2)
3 – 1 month	1163	29.4	(28.0, 30.8)
4 – >1 month	746	30.0	(28.2, 31.8)

### Access to surgery by list size

The percentage of patients receiving on-time surgery decreases with the list size from 40% (37–42%) in list-size category 1 (clearance time less than one week) to 30% (28–32%) in list-size category 4 (clearance time over one month), Table 2. Among all patients, the crude odds of on-time surgery was 58% lower in list-size category 2, OR = 0.42 (0.37–0.48), 66% lower in category 3, OR = 0.34 (0.31–0.38), and 71% lower in category 4, OR = 0.29 (0.25–0.32), compared to list-size category 1 (data not shown).

For semi-urgent and non-urgent patients, Table 3 shows the association between list size and the probability of on-time surgery as measured by unadjusted odds ratios. In the semi-urgent group, the crude odds of on-time surgery were 51% lower in list-size category 2, OR = 0.49 (0.42–0.57), 56% lower in category 3, OR = 0.44 (0.39–0.50), and 43% lower in category 4, OR = 0.57 (0.49–0.65).

**Table 3:** Association between wait-list size and chances of on-time surgery as measured by odds ratios derived from discrete-time survival regression models

	**Semi-urgent**		**Non-urgent**	
	**Crude**	Adjusted*	**Crude**	Adjusted*
**Surgery demand**	**OR (95% CI)**	**OR (95% CI)**	**OR (95% CI)**	**OR (95% CI)**
Wait-list size category				
1 – <1 week list-size	1.00	1.00	1.00	1.00
2 – half month list-size	0.49 (0.42, 0.57)	0.64 (0.54, 0.75)	0.88 (0.62, 1.25)	0.75 (0.51, 1.09)
3 – 1 month list-size	0.44 (0.39, 0.50)	0.53 (0.45, 0.62)	0.39 (0.28, 0.55)	0.38 (0.26, 0.56)
4 – >1 month list-size	0.57 (0.49, 0.65)	0.49 (0.41, 0.60)	0.30 (0.21, 0.41)	0.31 (0.20, 0.47)
Direct admission^†^	-	0.93 (0.91, 0.95)	-	0.90 (0.87, 0.94)

* Adjusted for age, sex, comorbidity, calendar period, hospital, and week on the list.

^† ^Associated with one additional surgery performed without wait-list registration

In the non-urgent group, the crude odds of on-time surgery were 12% lower in list-size category 1, OR = 0.88 (0.62–1.25), 61% lower in category 3, OR = 0.39 (0.28–0.55), and 70% lower in category 4, OR = 0.30 (0.21–0.41).

All urgent patients had a list-size category 1. Therefore the effect of the list size was examined using regression analysis.

### Regression analysis

In urgent patients wait-list size was studied as continuous variable ranging between 0 (28.1%) and 10 or more (1.7%). The effect of additional patient on the wait lists at registration was not significant, OR = 0.97 (0.86–1.10), after adjustment for age, sex, comorbidity, calendar period, hospital, and week on the list.

For semi-urgent and non-urgent patients, Table 3 shows the association between list size and the probability of on-time surgery as measured by the adjusted odds ratios. In the semi-urgent group, the odds of on-time surgery were 36% lower in list-size category 2, OR = 0.64 (0.54–0.75), 47% lower in category 3, OR = 0.53 (0.45–0.62), and 51% lower in category 4, OR = 0.49 (0.41–0.60), compared to list-size category 1 (clearance time less than one week).

In the non-urgent group, the odds of on-time surgery were 25% lower in list-size category 2, OR = 0.75 (0.51–1.09), 62% lower in category 3, OR = 0.38 (0.26–0.56), and 69% lower in category 4, OR = 0.31 (0.20–0.47), compared to list-size category 1.

Every time an additional patient was operated without being registered on wait lists, for non-urgent patients registered in that week the odds of on-time surgery were reduced by 10%, OR = 0.90 (0.87–0.94). Similarly, for semi-urgent patients the odds of on-time surgery were reduced by 7%, OR = 0.93 (0.91–0.95), and for urgent patients were reduced by 25%, OR = 0.75 (0.65–0.87).

## Discussion

Whether waiting times vary due to chance alone after adjustment for clinical factors and variations in demand remains an important question in health services research on access to care. However, chances of late surgery have not been previously described according to the length of wait list at registration in a multiple-list setting. This paper examines the relationship between the proportion of patients undergoing surgery within accepted standards and the length of the wait lists at registration for CABG surgery on multiple wait lists in a health system where all medically necessary services are publicly funded.

Using records from the provincial population-based registry of patients identified as needing isolated CABG, we determined the proportion of listed patients undergoing the operation within target access times across the list size categories. The list size was a simple count of patients with higher or equal priority present on the list at registration of a new patient on a given list.

We found that two in three patients were at risk of late surgery if registered on long wait lists. Our results provide evidence that list size had an effect on chances of undergoing elective surgery on time, with lower chances for longer lists in semi-urgent and non-urgent patients. When clearance time exceeded one week, half a month, or one month, the odds of on-time surgery were, respectively, 36%, 47%, and 51% lower in the semi-urgent group, and 25%, 62%, and 69% lower in the non-urgent group, compared to a shorter list, after adjustment for potential confounders. We also found an independent effect of the number of patients who were operated without being registered on wait lists.

Individual waiting times were analyzed as prospective observations beginning at the time of registration. We represented the wait for each patient by a sequence of binary indicators that indicate if the patient left the list at a certain wait-list week [13]. The likelihood function of such indicators can be factored into contributions that involve the conditional probabilities of surgery in a certain week among those remaining on the list. This observation justifies the practice of treating the binary indicators from one patient as independent Bernoulli trials [14]. To fit a pool of binary regression models developed for each patient by using the maximum likelihood method, it was assumed that binary indicators were independent across patients.

Misclassification of the recorded urgency of treatment is a concern in this analysis. Retrieved from the registry, the urgency category is a composite variable based on a variety of clinical factors. No audit has been performed to evaluate the quality of these records. The observation that higher priority patients were more likely to undergo CABG through the direct admission indicates that the degree of misclassification of priority was likely small. Another concern is that urgency of some patients were re-classified at the time of surgery. However, the timing of changes in urgency is not recorded.

The contribution of this paper to the research on access to care is three-fold. It provides evidence that for evaluation purposes one can not accurately estimate the proportion of late surgeries without considering the length of a wait list. It quantifies the effect of the queue length on the proportion of patients having late coronary bypass surgery in a network of hospitals with uniform standards for timing of surgery. It quantifies the effect of the operation performed without registration on a wait list, on the odds of on-time surgery in the patients registered on the list in that week.

## Conclusion

Chances of late surgery increase with the wait-list size for semi-urgent and non-urgent patients needing coronary bypass surgery. The weekly number of patients who move immediately from angiography to the operation without registration on a wait list reduced chances of surgery within target time in all urgency groups of listed patients. Our findings have implications for policies on access to elective cardiac surgery in a network of hospitals. If queue length varies substantially from hospital to hospital, policy makers may consider re-distribution of cases across hospitals with the aim of reducing the proportion of late surgeries. Our results also suggest that an informed decision on choosing a surgeon requires cardiologists and patients to consider information about the chance of undergoing surgery beyond a target time and associated risks. When projecting the expected time to treatment for patients who will be placed on the wait list, hospital managers should take into account the current list size as well as the weekly number of patients who require CABG immediately after undergoing coronary angiography. More research is needed to evaluate whether referral patterns across hospitals depend on wait-list size.

## Competing interests

The author(s) declare that they have no competing interests.

## Authors' contributions

*Study concept and design*: Sobolev. *Data acquisition*: Levy, Hayden. *Analysis and interpretation*: Sobolev, Kuramoto, Levy, Hayden. *Drafting of the manuscript*: Sobolev, Kuramoto.

## Pre-publication history

The pre-publication history for this paper can be accessed here:


